# The Genomic Architecture of Pregnancy-Associated Plasticity in the Maternal Mouse Hippocampus

**DOI:** 10.1523/ENEURO.0117-22.2022

**Published:** 2022-09-21

**Authors:** Alper Celik, Max Somer, Bharti Kukreja, Taiyi Wu, Brian T. Kalish

**Affiliations:** 1 Centre for Computational Medicine, Hospital for Sick Children, Toronto, Ontario, M5G 0A4, Canada; 2 Department of Biochemistry, University of Toronto, Toronto, Ontario, M5S 1A8, Canada; 3 Program in Neuroscience and Mental Health, SickKids Research Institute, Toronto, Ontario, M5G 0A4, Canada; 4 Department of Molecular Genetics, University of Toronto, Toronto, Ontario, M5S 1A8, Canada; 5 Division of Neonatology, Department of Paediatrics, Hospital for Sick Children, Toronto, Ontario, M5G 0A4, Canada; 6 Department of Immunology, University of Toronto, Toronto, Ontario, M5S 1A8, Canada

**Keywords:** epigenome, plasticity, pregnancy, single-cell genomics, transcriptome

## Abstract

Pregnancy is associated with extraordinary plasticity in the maternal brain. Studies in humans and other mammals suggest extensive structural and functional remodeling of the female brain during and after pregnancy. However, we understand remarkably little about the molecular underpinnings of this natural phenomenon. To gain insight into pregnancy-associated hippocampal plasticity, we performed single nucleus RNA sequencing (snRNA-seq) and snATAC-seq from the mouse hippocampus before, during, and after pregnancy. We identified cell type-specific transcriptional and epigenetic signatures associated with pregnancy and postpartum adaptation. In addition, we analyzed receptor-ligand interactions and transcription factor (TF) motifs that inform hippocampal cell type identity and provide evidence of pregnancy-associated adaption. In total, these data provide a unique resource of coupled transcriptional and epigenetic data across a dynamic time period in the mouse hippocampus and suggest opportunities for functional interrogation of hormone-mediated plasticity.

## Significance Statement

The female brain undergoes extraordinary plasticity during and after pregnancy, but the molecular mechanisms that regulate this hormone-responsive critical period are poorly understood. We performed an integrated analysis of single nucleus RNA-sequencing (snRNA-seq) and snATAC-seq before, during, and after pregnancy in the female mouse hippocampus. We identified candidate transcriptional and epigenetic regulators of maternal plasticity, including those implicated in neurogenesis, neurotransmission, and structural remodeling. Ligand-receptor analysis revealed signaling networks that are dynamically responsive to hormone levels during and after pregnancy. Our multiomics characterization provides a detailed landscape of complementary layers of regulatory control governing peripartum plasticity and identify candidate regulators of hormone-mediated adaptation in the maternal brain.

## Introduction

Pregnancy is a period of remarkable physiological adaptation in the maternal brain. Pregnancy-associated neuroplasticity is observed across mammals, with variations in temporal and regional specificity (Leuner and Sabihi, 2016). The profound molecular and cellular changes in the maternal brain are presumed to promote offspring survival and alter maternal behavior (Brunton and Russell, 2008), but these changes also have implications for maternal mental health. Critical periods for plasticity, like pregnancy, are also periods of vulnerability for neurologic disease. Pregnant and postpartum women are at increased risk for psychiatric disorders, including depression, anxiety, and psychosis (Hillerer et al., 2014). Therefore, insight into physiologic pregnancy plasticity may also shed light on potential mechanisms for pregnancy-associated mental illness.

Maternal brain plasticity is predominantly driven by dynamic hormone levels in the peripartum period, as pregnancy-associated neurologic changes have been reproduced in virgin female rodents through exogenous hormone treatment (Keyser-Marcus et al., 2001; Kinsley et al., 2006). Steroid hormones, which are in flux during and after pregnancy, markedly affect neurologic function via intracellular and membrane-bound steroid receptors expressed throughout the brain (Moraga-Amaro et al., 2018). Steroids such as progesterone regulate cell survival, synapse formation and dendritogenesis, whereas estrogens modulate synaptic plasticity and learning (Rossetti et al., 2016). Similarly, female sex steroids have notable effects in non-neuronal cells, such as promoting myelination, glutamate uptake in astrocytes, and the inflammatory action of microglia (Kipp et al., 2016). While the precise trajectory of hormone concentrations vary between rodents and humans, both progesterone and estradiol increase during gestation in mammals, ultimately peaking around parturition (Duarte-Guterman et al., 2019). Similarly, glucocorticoids follow an upward trend during pregnancy, and in mice, glucocorticoid levels increase dramatically from mid to late gestation (Solano and Arck, 2020).

There is a major gap in knowledge in the molecular underpinnings of adaptation in the brain during pregnancy. Given that pregnancy-associated plasticity is highly brain region-specific, we focus on the hippocampus because of its importance in postpartum maternal care behavior, as well as memory, mood, and stress resiliency (Duarte-Guterman et al., 2019). Neither the cell type-specific transcriptional nor *cis*-regulatory mechanisms underlying maternal brain plasticity have been previously explored. To begin to address this gap, we leveraged complementary single nuclei RNA-sequencing (snRNA-seq) and snATAC-seq of the maternal hippocampus before, during, and after pregnancy. We probe cell network ligand-receptor interactions during and after pregnancy and characterize enriched transcription factor (TF) motifs that define cell type identity and function in the murine hippocampus. This joint transcriptional and epigenetic atlas provides molecular support of pregnancy-associated structural remodeling of hippocampal architecture, as well as changes in neurogenesis and neurotransmission.

## Materials and Methods

### Tissue collection and generation of single-nuclei suspensions

All animal procedures were performed in accordance with the Harvard Medical School animal care committee’s regulations. Virgin female mice were euthanized at eight weeks of age. Pregnant mice were euthanized at embryonic day (E)18, and postpartum female mice were euthanized three weeks after parturition. The hippocampus was freshly dissected and flash frozen at −80°C. Frozen tissue was thawed in 500 μl buffer HB (0.25 m sucrose, 25 mm KCl, 5 mm MgCl2, 20 mm Tricine-KOH pH 7.8, 0.15 mm spermine tetrahydrochloride, 0.5 mm spermidine trihydrochloride, and 1 mm DTT). The tissue was transferred to a 2 ml dounce; 500 μl 5% IGEPAL CA-630 (Sigma) and 1 ml HB were added to the tissue, and the tissue was homogenized with a tight pestle 10–15 times. The sample was transferred to a 15 ml tube and total solution brought to 9 ml with HB. In a Corex tube (Fisher Scientific), 1 ml 30% iodixanol layered on top of 1 ml 40% iodixanol. The 9-ml sample was layered on top of the iodixanol cushion. The sample was spun at 10,000 × *g* for 18 min; 1 ml of sample at the 30–40% iodixanol interface was collected. After counting nuclei with a hemocytometer, the sample was diluted to 100,000 nuclei/ml with 30% iodixanol (with Rnasin) and subjected to single nuclear droplet encapsulation with inDrop (Klein et al., 2015).

### snRNA-seq and analysis

Individual nuclei were captured and barcoded using the inDrop platform as previously described (Klein et al., 2015). Briefly, single-cell suspensions were fed into a microfluidic device that packaged the cells with barcoded hydrogel microspheres and reverse transcriptase/lysis reagents. After cell encapsulation, primers were photo-released by UV exposure. Two technical replicate libraries were collected for each sample. Indexed libraries were pooled and sequenced on a Nextseq 500 (Illumina) to an average depth of ∼30,000 reads/nucleus. Sequencing data were aligned to the genome and processed according to a previously published pipeline (https://github.com/indrops/indrops). Unique molecular identifiers (UMIs) were used to link sequence reads back to individual captured molecules. All steps of the pipeline were run using default parameters.

All data processing was performed using Seurat (v. 4.0). Raw data were log normalized and scaled using default values. Variable features and principal components were then calculated using default values. Uniform manifold approximation and projection (UMAP) and t-distributed stochastic neighbor embedding (tSNE) dimensionality reductions were performed with default values. Batch effects were not evident in the dimensionality reductions, and therefore, the data were then analyzed as-is without further corrections.

### Identification of cell types in snRNA-seq

We used scPred (Alquicira-Hernandez et al., 2019) to train radial basis kernel support vector machines (SVMs) in a one versus all fashion using data deposited to Allen Brain Atlas (Yao et al., 2021). For consistency, we only used cells from the hippocampus (212,939 cells) to train the predictor models. Before training, the training data were independently preprocessed in the same fashion as above. For each cell type annotated in the Allen Brain Atlas, we trained a separate one versus all SVM model with a Radial Basis Function kernel. The training data were split 80/20; 80% of the training data were centered, scaled, and normalized in the same fashion as our dataset. Models were trained using a 5-fold cross validation approach and only models with area under the receiver operating characteristic (AUROC) > 0.95 were used. Using the remaining 20% of the training dataset, we then predicted the cell types of each cell in our dataset. Only cells with in-class prediction (e.g., CA1 vs all others) probability >0.95 were retained. We manually confirmed cell types based on known marker gene expression. Identified cell types (as some cell types corresponded to multiple clusters) with <100 cells per condition were not used for further analysis.

### Differential expression and ligand-receptor analysis

For each identified cell type, we performed differential expression analysis using Seurat with MAST method between different conditions (e.g., virgin CA3 vs E18 CA3). We used Benjamini–Hochberg corrected *p*-values for significance. Genes with <5% false discovery rate (FDR) met statistical criteria for significant differential expression. Ligand-receptor analysis was performed using CellChat (version 1.1.3) with default parameters (Jin et al., 2021).

### snATAC-seq

Tissue was processed for single nucleus suspensions as described above. Nuclei were processed using the 10× Chromium scATAC-seq kit (v1.0) per manufacturer’s instructions. Libraries were sequenced to an average depth of ∼50,000 reads/nucleus. Sequencing reads were demultiplexed and aligned to the mm10 genome using CellRanger with default parameters. Resulting outputs were then processed using Signac (v 1.5; Stuart et al., 2021). Low quality cells were removed using filters peak region fragments <100,000, percent reads in peaks >40, blacklist site ratio <0.025, nucleosome signal <4, and transcriptional start site (TSS) enrichment score >2. Remaining preprocessing and dimensionality reduction was performed according to Signac recommendations using default parameters.

Cell types were determined using the snRNA-seq data labels based on Signac recommendations (Stuart et al., 2021). We used FindTransferAnchors and TransferData functions to annotate cell types. We then again checked for consistency between unsupervised clustering and label transfer. Similar to snRNA-seq data, clusters with high cell type heterogeneity were discarded and cell types were assigned to clusters based on the majority of the labels. Cell types with <100 cells per condition were not analyzed further.

### Differential accessibility and TF motif analysis

Using cell type labels described above, we performed differential accessibility analysis using Signac’s FindMarkers function with log fold change threshold 0.1 and n_count_peaks as a latent variable. FDR <5% was used as the statistical significance threshold. Motif analysis was done using RunChromVAR function in Signac with default parameters. For differential motif activity, we used log2 fold change threshold of 0.1 with a 0 pseudocount. For statistical significance, we used an FDR threshold of 5%.

### Integrative analysis with ArchR

In order to perform co-accessibility and peak-to-gene linkage analysis, we processed snATAC-seq data with ArchR (Granja et al., 2021). Data were processed using default parameters. We used the addCoAccessibility and getCoAccessibility functions to generate co-accessibility data frames and figures. We performed peak-to-gene linkage analysis using the addPeak2GeneLinks function, and the plotPeak2GeneHeatmap function was used to generate a heatmap of peak-to-gene results.

### Data accessibility

Raw and processed sequencing data have been deposited in the Gene Expression Omnibus (GEO) under accession number GSE198447.

## Results

### Cell type-specific transcriptomic landscape of the maternal hippocampus

**Figure 1.**
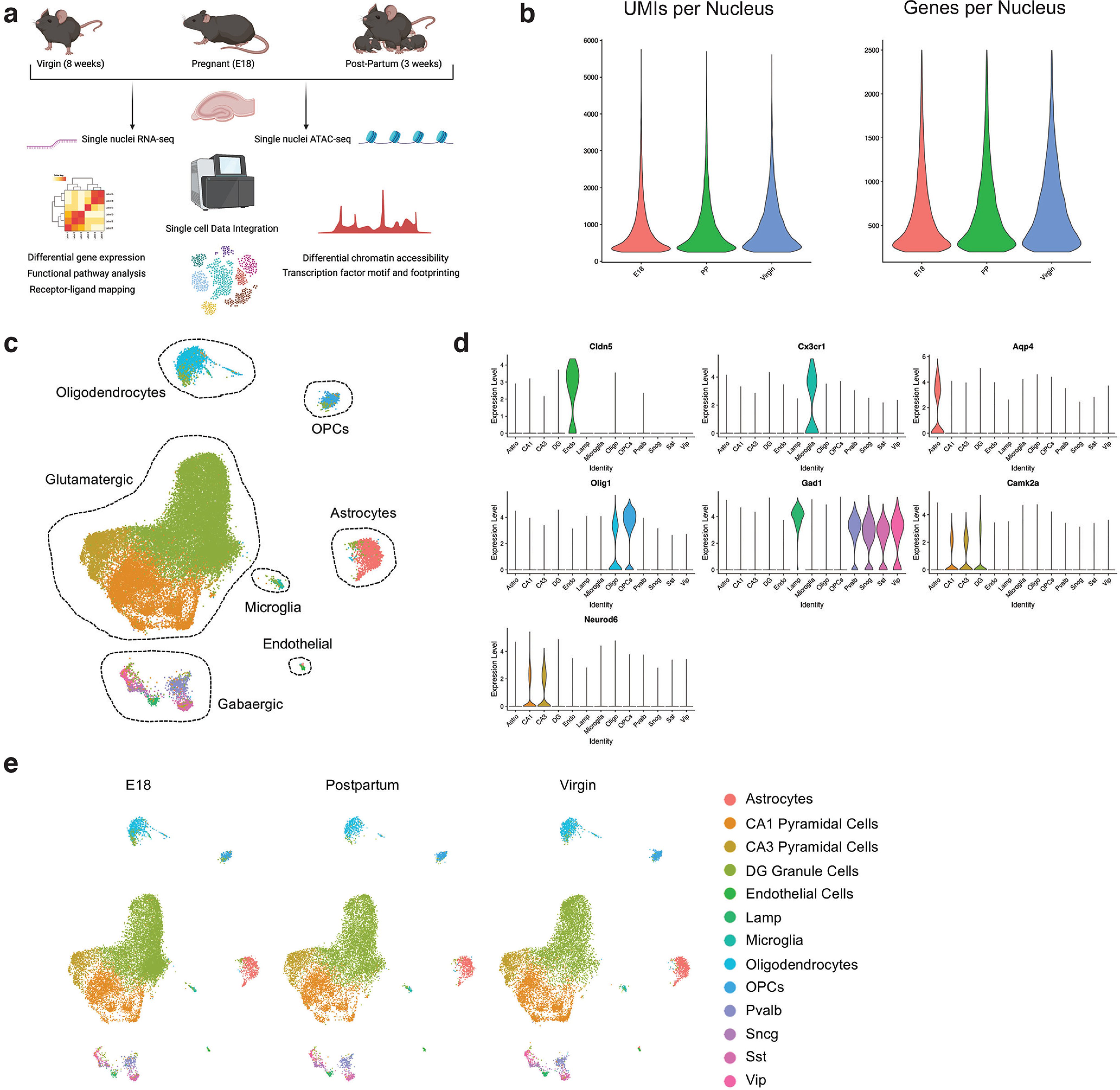
Single-cell investigation of the female hippocampus before, during, and after pregnancy. ***a***, Experimental schematic of snRNA-seq and snATAC-seq of the female hippocampus in virgin, pregnant (E18), and postpartum mice. ***b***, Quality control (QC) plots of UMIs and genes per nucleus in nuclei included in the final snRNA-seq dataset. ***c***, UMAP of snRNA-seq data with major cell types identified. Extended Data Figure 1-1 provides AUROC, sensitivity, and specificity data for SVM model for cell type identification in snRNA-seq. ***d***, Violin plots of canonical marker gene expression across cell types, as measured by snRNA-seq. ***e***, UMAPs of nuclei included in snRNA-seq dataset, separated by condition.

To identify cell type-specific programs governing plasticity during pregnancy, we performed droplet-based snRNA-seq from the female hippocampus under three conditions: eight-week-old virgin female mice, E18 pregnant female mice, and P21 (postpartum) female mice (Fig. 1*a*). These time points were selected to span the continuum of hormonal shifts that occur before, during, and after pregnancy. We analyzed three biological replicates at each time point, for a total of nine independent samples. Virgin mice were synchronized in their estrous cycle before tissue collection.

10.1523/ENEURO.0117-22.2022.f1-1 Extended Data Figure 1-1 Table of AUROC values by cell type for SVM model for cell type identification in snRNA-seq. The table displays the cell type, number of cells (*n*), number of features in the model, model type, AUROC value, sensitivity, and specificity. Download Figure 1-1, XLS file.

Nuclei were isolated from frozen tissue (see Materials and Methods) and processed using the inDrop platform (Klein et al., 2015). After computational filtering of low quality and doublet nuclei, we profiled a total of 40,652 nuclei by snRNA-seq. Quality control metrics are displayed in Figure 1*b*. We used the R package Seurat to perform unsupervised graph-based clustering of all nuclei, followed by cluster visualization with UMAP, a nonlinear dimensional reduction algorithm. To aid in the confident identification of cell types, we leveraged previously published high quality datasets from Allen Brain Atlas (Alquicira-Hernandez et al., 2019). With these expert labeled datasets, we used scPred (Alquicira-Hernandez et al., 2019) to train one versus all SVM models with radial kernels to identify cell types. Models were trained using a 5-fold cross-validation approach. Using each of the models, we then predicted the cell types of each cell in our dataset. Only cells with in-class prediction (e.g., CA1 pyramidal neurons versus all others) probability >0.95 were retained. Complete AUROC values are provided in Extended Data Figure 1-1.

**Figure 2.**
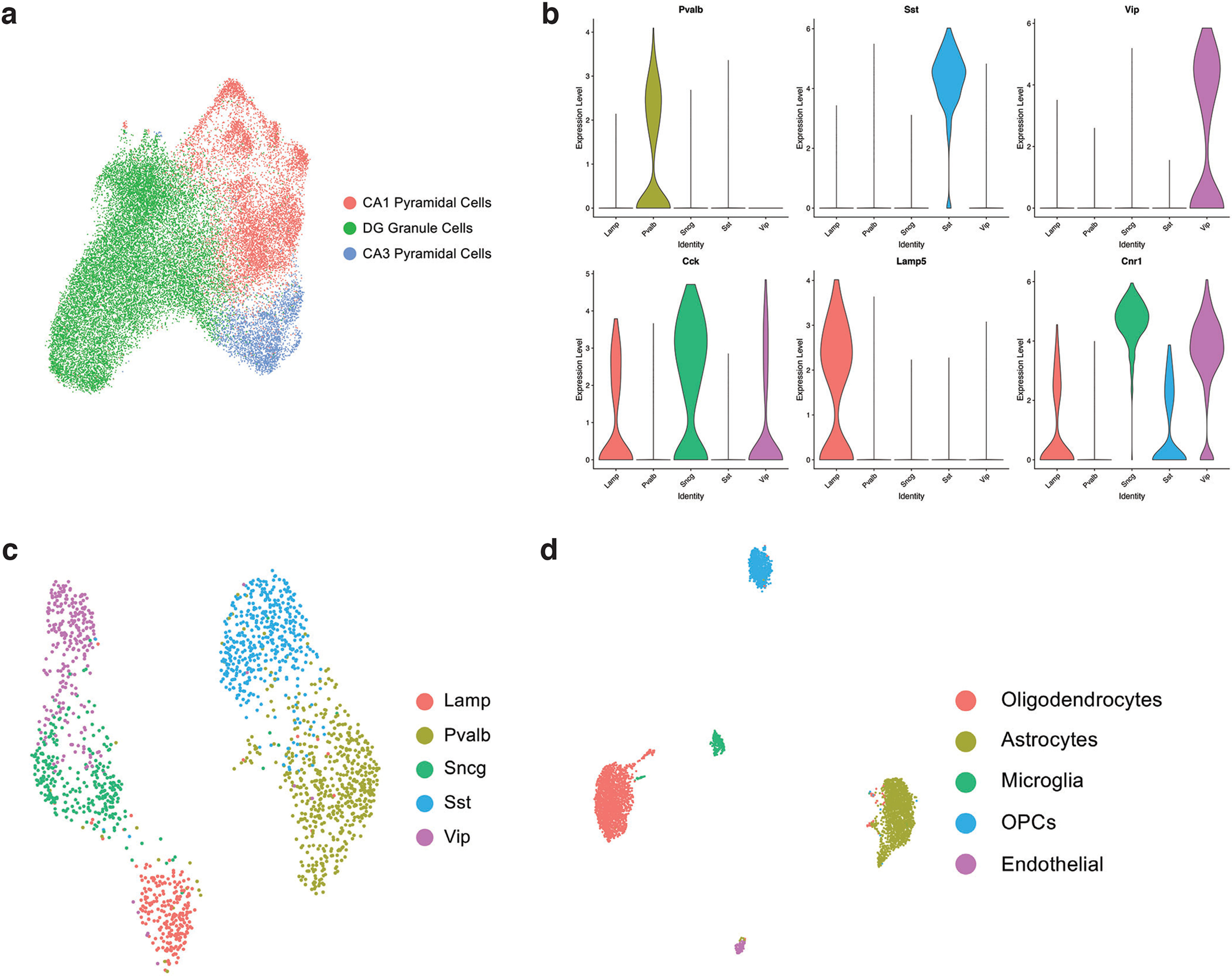
Subclustering analysis of snRNA-seq of the female hippocampus before, during, and after pregnancy. ***a***, UMAP of glutamatergic cells, with DG granule cells, CA1 pyramidal cells, and CA3 pyramidal cells labeled by color. ***b***, Violin plots of canonical interneuron marker genes, as measured by snRNA-seq. ***c***, UMAP of GABAergic cells, with interneuron subtypes labeled by color. ***d***, UMAP of non-neuronal cells, with subtypes labeled by color.

In total, we identified clusters corresponding to major hippocampal cell types: glutamatergic (granule cells, pyramidal cells), GABAergic, and non-neuronal (microglia, astrocytes, oligodendrocytes; Fig. 1*c*). Expression of canonical cell type marker genes affirmed cell identifies (Fig. 1*d*). Cell cluster assignment did not globally differ between conditions, suggesting that no new cell types were detected between the three conditions (Fig. 1*e*). Hippocampal cell types were subclustered into glutamatergic, GABAergic, and non-neuronal groups (Fig. 2*a–d*).

**Figure 3.**
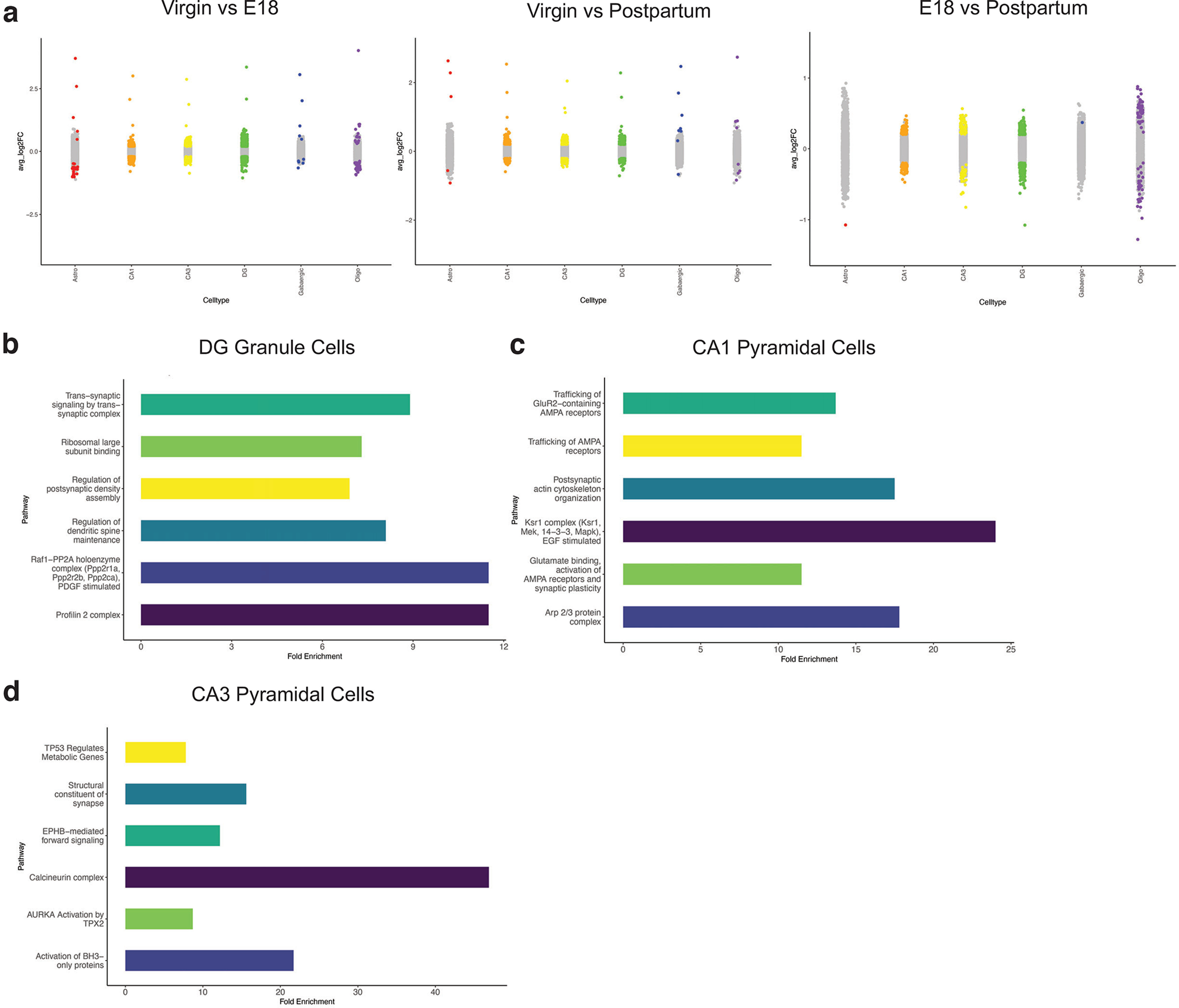
Cell type-specific differential gene expression analysis across the peripartum period. ***a***, Strip plots of average log2 fold change by cell type. Colored dots indicate genes with statistically significant changes (FD < 5%) between conditions. Extended Data Figure 3-1 provides complete differential gene expression results. ***b***, GO analysis of differentially expressed genes (FDR < 5%) between virgin and E18 in DG granule cells. Extended Data Figure 3-2 provides complete results of GO analysis. ***c***, GO analysis of differentially expressed genes (FDR < 5%) between virgin and E18 in CA1 pyramidal cells. Extended Data Figure 3-2 provides complete results of GO analysis. ***d***, GO analysis of differentially expressed genes (FDR < 5%) between virgin and E18 in CA3 pyramidal cells. Extended Data Figure 3-2 provides complete results of GO analysis.

We implemented differential gene expression analysis to investigate transcriptomic changes across pregnancy within each cell type. Statistical significance was assigned to genes with a FDR < 5%. All differential gene expression output is summarized by cell type in Extended Data Figure 3-1. We identified cell type-specific changes in gene expression across the time course of pregnancy and postpartum period (Fig. 3*a*). We performed gene ontology (GO) analysis of statistically significant differentially expressed genes between timepoints to identify functional pathways that exhibit peripartum cell type-specific changes (Fig. 3*b–d*; Extended Data Fig. 3-2). Regulation of the actin cytoskeleton (including the profilin complex), endocytosis, trans-synaptic signaling, and the trafficking of neurotransmitter receptors were commonly identified pathways, consistent with hippocampal plasticity during this critical window.

10.1523/ENEURO.0117-22.2022.f3-1 Extended Data Figure 3-1 Table of differential gene expression results from snRNA-seq between virgin, E18, and postpartum conditions. Each tab of the file contains results of differential gene expression analysis in a specific cell type, organized by pairwise comparison between conditions (virgin, E18, and postpartum). The results list the gene of interest, *p*-value, adjusted *p*-value, average log2 fold change, FDR, pairwise comparison, and cell type. Download Figure 3-1, XLS file.

10.1523/ENEURO.0117-22.2022.f3-2 Extended Data Figure 3-2 GO analysis of statistically significant differentially expressed genes in the virgin versus E18 comparison. Each tab of the file contains results of GO analysis by select cell type. Only statistically significant GO pathways are listed. Download Figure 3-2, XLS file.

We then focused our interpretation of the snRNA-seq data on three specific themes, neurogenesis, neurotransmission, and structural remodeling, that are known to be perturbed in the female brain during and after pregnancy (Galea et al., 2014; Hillerer et al., 2014; Duarte-Guterman et al., 2019).

#### Neurogenesis

The effect of pregnancy on the birth of new neurons demonstrates temporal, region, and species specificity (Leuner and Sabihi, 2016). In the maternal mouse brain, hippocampal neurogenesis is decreased at E18 relative to age-matched nonpregnant controls (Kim et al., 2010). This reduction in hippocampal neurogenesis is sustained following parturition, with the number of neural precursor cells (NPCs) returning to prepregnancy levels three weeks postpartum (Rolls et al., 2008). Thus, fluctuations in neurogenesis might contribute to the reductions in hippocampus volume that occur in pregnancy in both rats and humans (Galea et al., 2000; Hoekzema et al., 2017).

**Figure 4.**
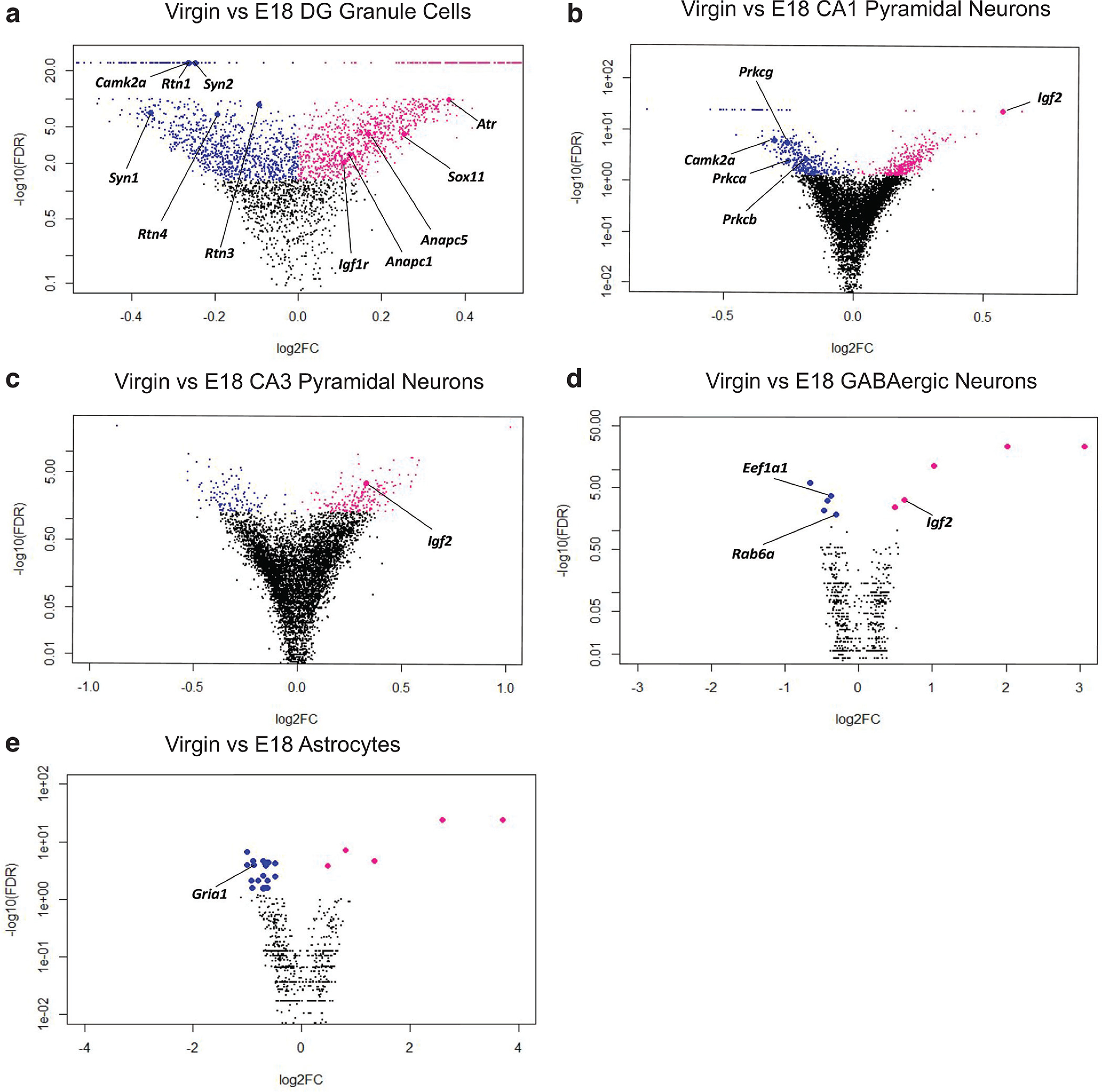
Differential gene expression analysis to identify candidate regulators of peripartum plasticity. ***a***, Volcano plot of differential gene expression between virgin and E18 condition in DG granule cells. Statistically significant genes (FDR < 5%) are colored, with blue indicating lower expression and pink indicating higher expression at E18. ***b***, Volcano plot of differential gene expression between virgin and E18 condition in CA1. Statistically significant genes (FDR < 5%) are colored, with blue indicating lower expression and pink indicating higher expression at E18. ***c***, Volcano plot of differential gene expression between virgin and E18 condition in CA3. Statistically significant genes (FDR < 5%) are colored, with blue indicating lower expression and pink indicating higher expression at E18. ***d***, Volcano plot of differential gene expression between virgin and E18 condition in GABAergic cells. Statistically significant genes (FDR < 5%) are colored, with blue indicating lower expression and pink indicating higher expression at E18. ***e***, Volcano plot of differential gene expression between virgin and E18 condition in astrocytes. Statistically significant genes (FDR < 5%) are colored, with blue indicating lower expression and pink indicating higher expression at E18.

The dentate gyrus (DG) is the site of hippocampal neurogenesis. We probed our snRNA-seq dataset of the hippocampus and identified gene expression patterns in DG granule cells consistent with decreased neurogenesis during late pregnancy. Specifically, we found reduced expression of the transcriptional activator *Sox11* at E18 compared with virgin mice (Fig. 4*a*). *Sox11* is highly expressed in regions of adult neurogenesis and is sufficient to induce the differentiation of NPCs to immature neurons (Haslinger et al., 2009). Notably, the expression of Neurite outgrowth inhibitor (*Nogo*), also known as Reticulon 4, is increased at E18 relative to virgin mice in DG granule cells (Fig. 4*a*). This is consistent with the role of Nogo and its receptor NgR1 as negative regulators of NPC proliferation (Rolando et al., 2012). We found that the expression of other reticulon family members, including Reticulon (*Rtn*) 1 and 3, also exhibit higher expression in DG granule cells during pregnancy when compared with virgin levels (Fig. 4*a*). Reticulon family members are known to promote apoptosis (Yang and Strittmatter, 2007) which is notable given the importance of apoptosis in shaping cell fate during neurogenesis (Tashiro et al., 2006; Sierra et al., 2010).

We also identified coordinate changes in insulin-like growth factor signaling in the hippocampus during pregnancy. Insulin-like growth factor 2 (*Igf2*) expression in CA1 and CA3 pyramidal neurons, as well as in GABAergic interneurons, is lower during pregnancy relative to virgin controls (Fig. 4*b–d*). In parallel, insulin-like growth factor 1 receptor (*Igf1r*) expression is also lower during pregnancy in the DG (Fig. 4*a*). IGF2 primarily binds to IGF1R and regulates cell proliferation during adult hippocampal neurogenesis (Bracko et al., 2012; Ferrón et al., 2015). Specifically, IGF2 in NPCs stimulates self-renewal and increases mRNA expression of the canonical stem cell TFs Oct4 and Sox1 (Ziegler et al., 2012).

We further identified lower expression of cell cycle regulators in DG granule cells of pregnant mice relative to virgin controls, including three subunits of the anaphase-promoting complex (*Anapc1*, *Anapc5*, and *Anapc7*) and ataxia telangiectasia and Rad3 related (*Atr*; Fig. 4*a*). In mouse embryos, ANAPC facilitates neurogenesis, and ATR governs the transition from G_2_ to M in the mitotic cycle of NPCs (Delgado-Esteban et al., 2013; Enriquez-Rios et al., 2017).

#### Neurotransmission

In both humans and rodents, memory function is profoundly altered in the peripartum and postpartum periods (Pawluski et al., 2016). Given that memories are stored through enhanced connectivity between groups of neurons, changes in neurotransmission may underlie modifications in memory by affecting synaptic strength (Josselyn et al., 2015). For example, in rats, motherhood induces long-term potentiation (LTP) with higher NMDA and non-NMDA-mediated hippocampal neurotransmission relative to virgin controls (Lemaire et al., 2006).

Accordingly, we analyzed our snRNA-seq dataset for molecular mediators of neurotransmission. We found that several conventional protein kinase C isozymes, including *α*, *β*, and *γ*, have increased expression in DG granule cells and CA1 pyramidal neurons during pregnancy compared with virgin mice (Fig. 4*a*,*b*). PKCα is the requisite isozyme for structural LTP, and PKCγ is likely connected to LTP through its phosphorylation of the NMDA receptor (NMDAR) subunit NR1 (Gomis-González et al., 2021). We identified further changes in gene expression related to LTP in the hippocampus during pregnancy. Specifically, at E18 relative to virgin, we noted increased expression of the *ɑ* and *β* isoforms of calmodulin-dependent protein kinase II (*CaMKII*) in DG granule cells and CA1 pyramidal neurons (Fig. 4*a*,*b*). This serine/threonine-protein kinase is a vital component of LTP by modulation of AMPAR insertion and conductance (Lisman et al., 2012). Consistent with increased CaMKII, we identified the increased expression of Synapsin 1 (*Syn1*) and Synapsin 2 (*Syn2*) in DG granule cells at E18 relative to virgin (Fig. 4*a*). These phosphoproteins are subject to CAMKII activation and have many neuronal functions, including linking vesicles to the cytoskeleton at the presynaptic terminal (Cesca et al., 2010). Syn1 deficient mice have impaired glutamate release (L Li et al., 1995), and likewise, GABA neurotransmission is distorted in Syn2 deficient mice (Medrihan et al., 2013). We also found increased expression of Ras-related protein Rab-3A (*RAB3A*) and Rab-3C (*RAB3C*) in DG granule cells at E18 relative to virgin (Fig. 4*a*), consistent with changes in the presynaptic terminal. These small GTP binding proteins control calcium-induced exocytosis (Schlüter et al., 2004) and BDNF-dependent glutamate release (Simsek-Duran and Lonart, 2008).

Non-neuronal cells, particularly astrocytes, are essential regulators of neuronal communication because of their ability to sense and respond to neurotransmitter release (Hwang et al., 2021). In astrocytes, we found increased expression of glutamate receptor ionotropic AMPA type subunit 1 (*Gria1*) in pregnant mice relative to virgin controls (Fig. 4*e*). Astrocytes regulate glutamate homeostasis through glutamate uptake, enabling them to modulate neighboring neuronal activity (Droste et al., 2017; Mahmoud et al., 2019).

#### Structural remodeling

The maternal neural circuit architecture exhibits substantial structural plasticity during pregnancy (Hillerer et al., 2014). Notably, membrane-bound estrogen receptors are prevalent in the mammalian hippocampus and act through a defined mechanism to mediate dendritic spine as well as synapse formation (Hara et al., 2015). In our data, we identified differential expression of genes implicated in structural remodeling during and after pregnancy.

Strikingly, we found numerous candidates pertinent to actin restructuring to be more highly expressed during pregnancy. This is relevant because dendritic spines have a complex cytoskeleton of branched and linear actin filaments (Basu and Lamprecht, 2018). Consequently, dendritic size and morphology regulation, such as in response to glutamate stimulation, requires tightly controlled actin polymerization and depolymerization (Matsuzaki et al., 2004; Borovac et al., 2018). The expression of Actin Depolymerizing Factor (*ADF*) is higher during pregnancy in DG granule cells compared with virgin controls (Fig. 4*a*). The ADF/Cofilin family governs the structural plasticity of dendritic spines, and active ADF/Cofilin enables increases in spine size during LTP and the diminishing of spines associated with LTD (Rust, 2015). Similar to ADF/Cofilin, the Rho GTPase family modifies dendrite morphology through actin skeleton dynamics (Murakoshi et al., 2011). In DG granule cells, we noted increased expression of *RhoB* and *Rac1* at E18 relative to virgin mice (Fig. 4*a*). Consistent with this RhoGTPase mechanism, we identified increased expression of p21 activated kinase (*Pak1*) at E18 relative to virgin in DG granule cells (Fig. 4*a*). Pak1 is a notable downstream serine/threonine kinase effector of the Rho GTPase family, and its phosphorylation is associated with neurite organization and outgrowth (Rashid et al., 2001; Nikolić, 2008).

Moreover, in CA1 pyramidal neurons and DG granule cells during pregnancy, we found increased expression of the postsynaptic density protein 95 (*PSD-95*) and synapse-associated protein 102 (*SAP-102*) compared with postpartum mice (Fig. 4*a*,*b*). Together, these membrane-associated guanylate kinases (MAGUKs) are critical structural components of the postsynaptic density (PSD) and serve to regulate its size (Zheng et al., 2011). Interestingly, the translation of PSD-95 mRNA is stimulated by estrogen signaling, and this process is thought to partly underlie estrogen’s positive effect on synaptogenesis (Akama and McEwen, 2003).

### Ligand-Receptor analysis of the maternal hippocampus

**Figure 5.**
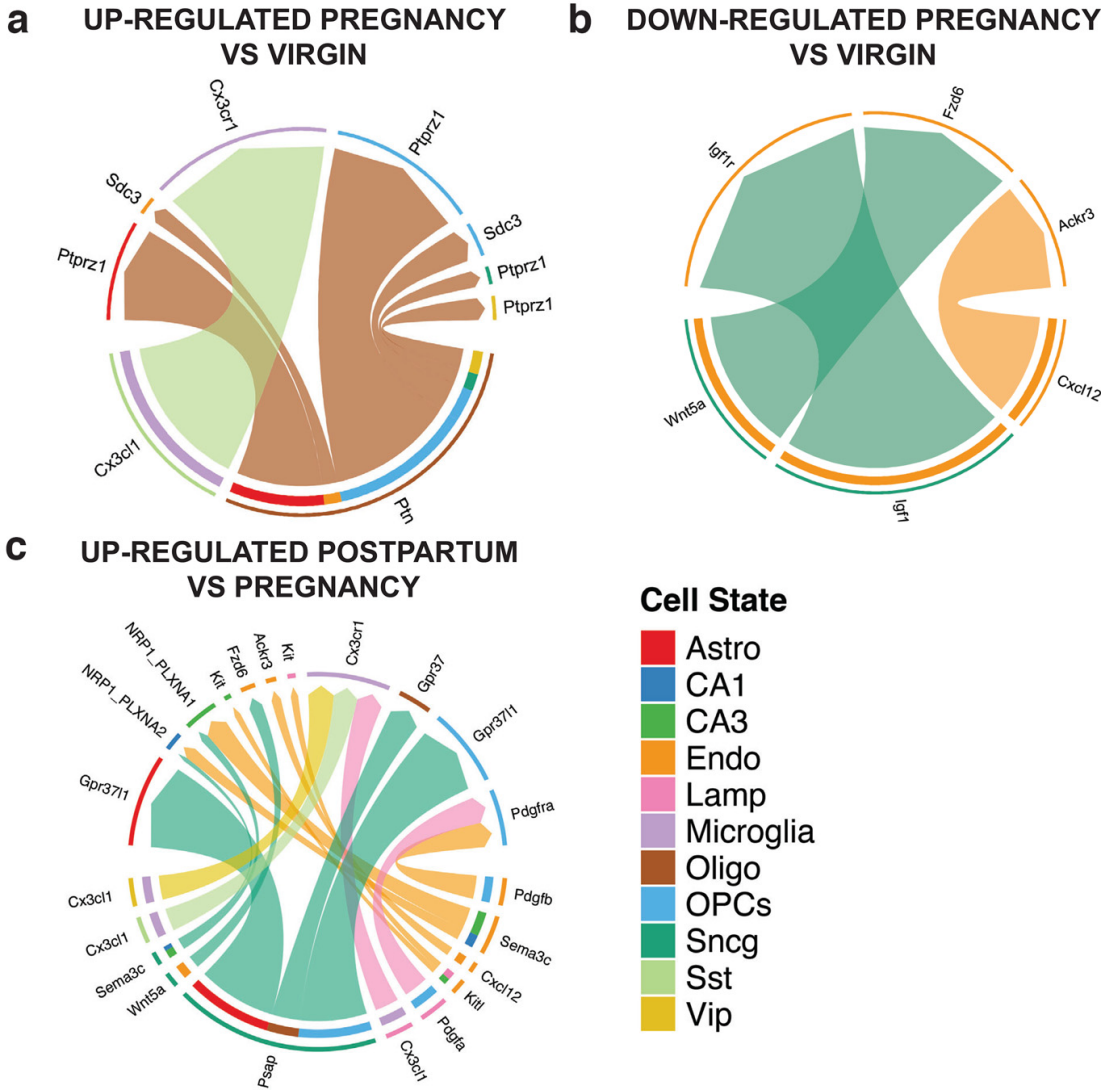
Ligand-receptor analysis of snRNA-seq reveals dynamic signaling networks during pregnancy. ***a***, Chord diagram of cell type-specific signaling pathways that are upregulated at E18 compared with virgin. Cell type identity of the “sending” cell is indicated in the outermost edge of the diagram, while cell identity of the “receiving” cell is indicated by the color second from the outside. Colored arrows in the diagram indicate specific ligand/receptor pathways. ***b***, Chord diagram of cell type-specific signaling pathways that are downregulated at E18 compared with virgin. Cell type identity of the “sending” cell is indicated in the outermost edge of the diagram, while cell identity of the “receiving” cell is indicated by the color second from the outside. Colored arrows in the diagram indicate specific ligand/receptor pathways. ***c***, Chord diagram of cell type-specific signaling pathways that are upregulated postpartum compared with pregnancy. Cell type identity of the “sending” cell is indicated in the outermost edge of the diagram, while cell identity of the “receiving” cell is indicated by the color second from the outside. Colored arrows in the diagram indicate specific ligand/receptor pathways.

Cells in the brain communicate through soluble and membrane-bound components. To map ligand-receptor networks in the maternal hippocampus at single cell resolution, we used the open-source R toolkit CellChat (Jin et al., 2021). We identified several signaling networks in the hippocampus that are perturbed during or after pregnancy. The CX3C signaling pathway is upregulated during pregnancy relative to virgin conditions (Fig. 5*a*). CX3C signaling regulates neuronal network maintenance and is dependent on interactions between the transmembrane chemokine CX3CL1 and its G-protein coupled receptor CX3CR1, found only in microglia (Cardona et al., 2006; Paolicelli et al., 2011; Pawelec et al., 2020). The dominant interaction occurs between the CX3CR1 on microglia and fractalkine secreted by somatostatin interneurons. Thus, we posit that a method of synapse refinement during pregnancy involves microglial phagocytosis driven by the CX3C pathway. Alternatively, CX3C signaling might underlie the increased oligodendrocyte differentiation during pregnancy (Gregg, 2009), as interneurons mediate developmental oligodendrogenesis through secretion of CX3CL1 (Voronova et al., 2017).

We also found that the pleiotrophin (PTN) network is upregulated during pregnancy compared with both virgin and postpartum conditions (Fig. 5*a*). Interactions between PTN with PTPRZ receptors on oligodendrocyte precursor cells (OPCs) stimulate differentiation to oligodendrocytes, and therefore the capacity for myelination might be increased during pregnancy (Tanga et al., 2019). This is consistent with murine lineage-tracing experiments that indicate a 74% increase in oligodendrocyte formation in the corpus callosum at E18 relative to virgin controls (Gregg et al., 2007).

Three signaling networks (CXCL12/Ackr3, IGF1/IGF1r, Wnt5a/Fzd6) were significantly downregulated in pregnancy (E18) compared with virgin (Fig. 5*b*). Signaling between chemokine CXCL12 and its receptor ACKR3 on endothelial cells declined in pregnancy but increased postpartum. CXCL12/ACKR3 signaling is important in directing cell migration and mediating angiogenesis (Guyon, 2014; Quinn et al., 2018). Similarly, IGF1/IGF1R signaling declined during pregnancy, which is notable given that IGF1 deficiency in the hippocampus is linked to depression (Torres Aleman, 2005; Mitschelen et al., 2011) Wnt5a signaling in endothelial cells also declined during pregnancy compared with virgin (Fig. 5*b*). Wnt ligands are important for angiogenesis, tight junction formation, and blood brain barrier integrity, which is notable given changes in permeability of the blood brain barrier during pregnancy (Daneman et al., 2009; Cipolla et al., 2011).

The Prosaposin (PSAP)/GPR37 signaling network increases postpartum compared with pregnancy (E18; Fig. 5*c*). PSAP and its receptors (GPR37 and GPR37l1) are expressed in astrocytes, OPCs, and oligodendrocytes. GPR37l1 signaling inhibits glutamate uptake in astrocytes and reduces neuronal NMDAR activity (Jolly et al., 2018). Loss of GPR37 in oligodendrocytes accelerates differentiation and hypermyelination, and therefore, lower levels of PSAP/GPR37 signaling may support increased myelination during pregnancy (Yang et al., 2016; Smith et al., 2017)

### *Cis*-regulatory landscape of the maternal hippocampus

**Figure 6.**
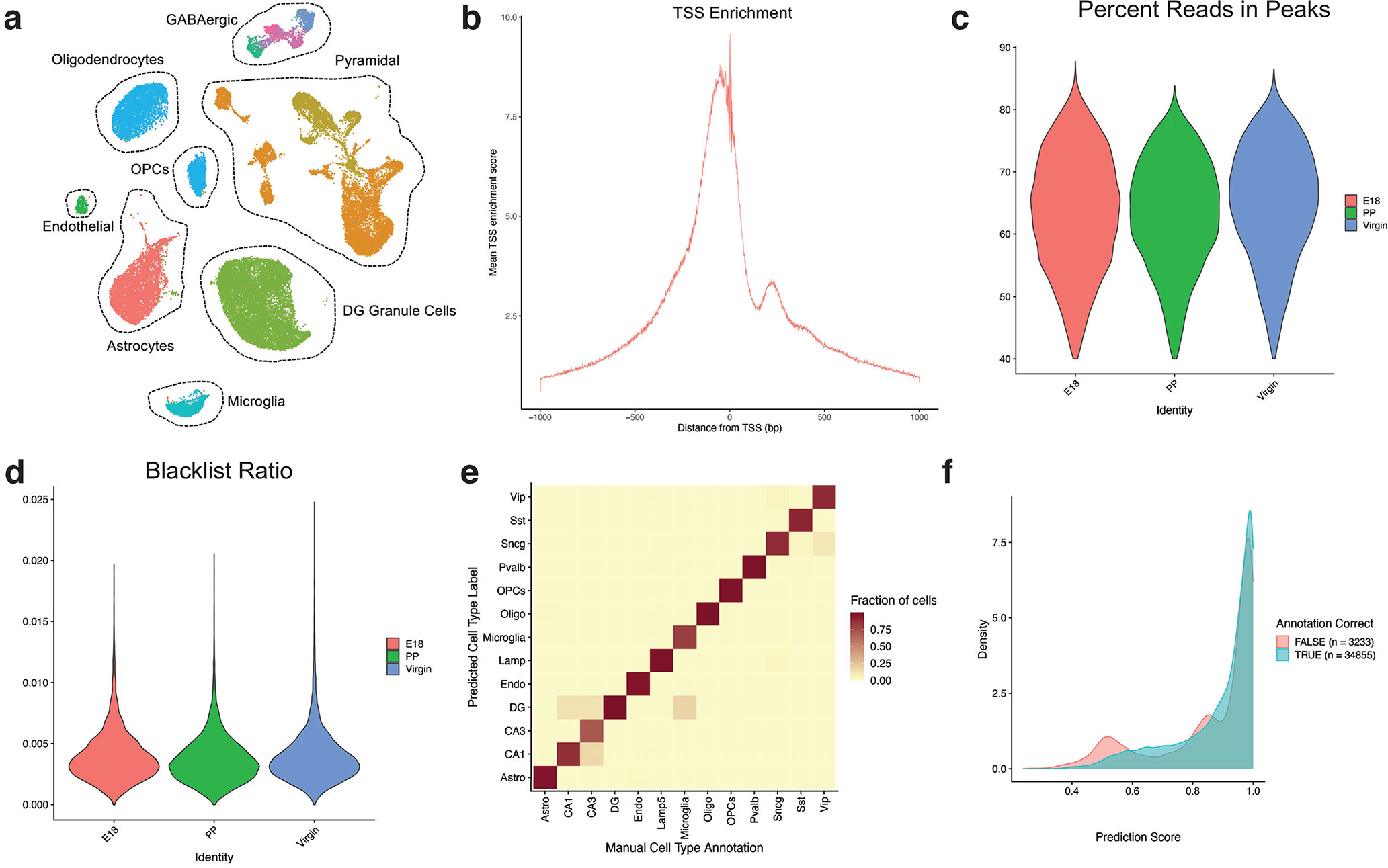
Clustering and quality control metrics of snATAC-seq of the murine hippocampus before, during, and after pregnancy. ***a***, UMAP of snATAC-seq data with major cell types identified. ***b***, TSS enrichment score, defined as the ratio of fragments centered at the TSS to fragments in TSS-flanking regions, for all cells included in snATAC-seq analysis, grouped by condition. ***c***, Percentage of reads in peaks for all cells included in snATAC-seq analysis, grouped by condition. ***d***, Ratio of reads in genomic blacklist regions as defined by the ENCODE project, representing reads that are often associated with artefactual signal. ***e***, Agreement between predicted snATAC-seq cell types, as identified by Seurat label transfer, and supervised (manual) cell type annotation based on the gene activity matrix of canonical cell type-specific marker genes. The color of the squares indicates the fraction of cells in which there is agreement in cell type identity. ***f***, Prediction score of label transfer between snRNA-seq and snATAC-seq, comparing nuclei with “true” identity assignment (concordance between Seurat-based label transfer analysis and manual annotation based on marker genes) and “false” identity assignment (disagreement between Seurat-based label transfer analysis and manual annotation based on marker genes).

**Figure 7.**
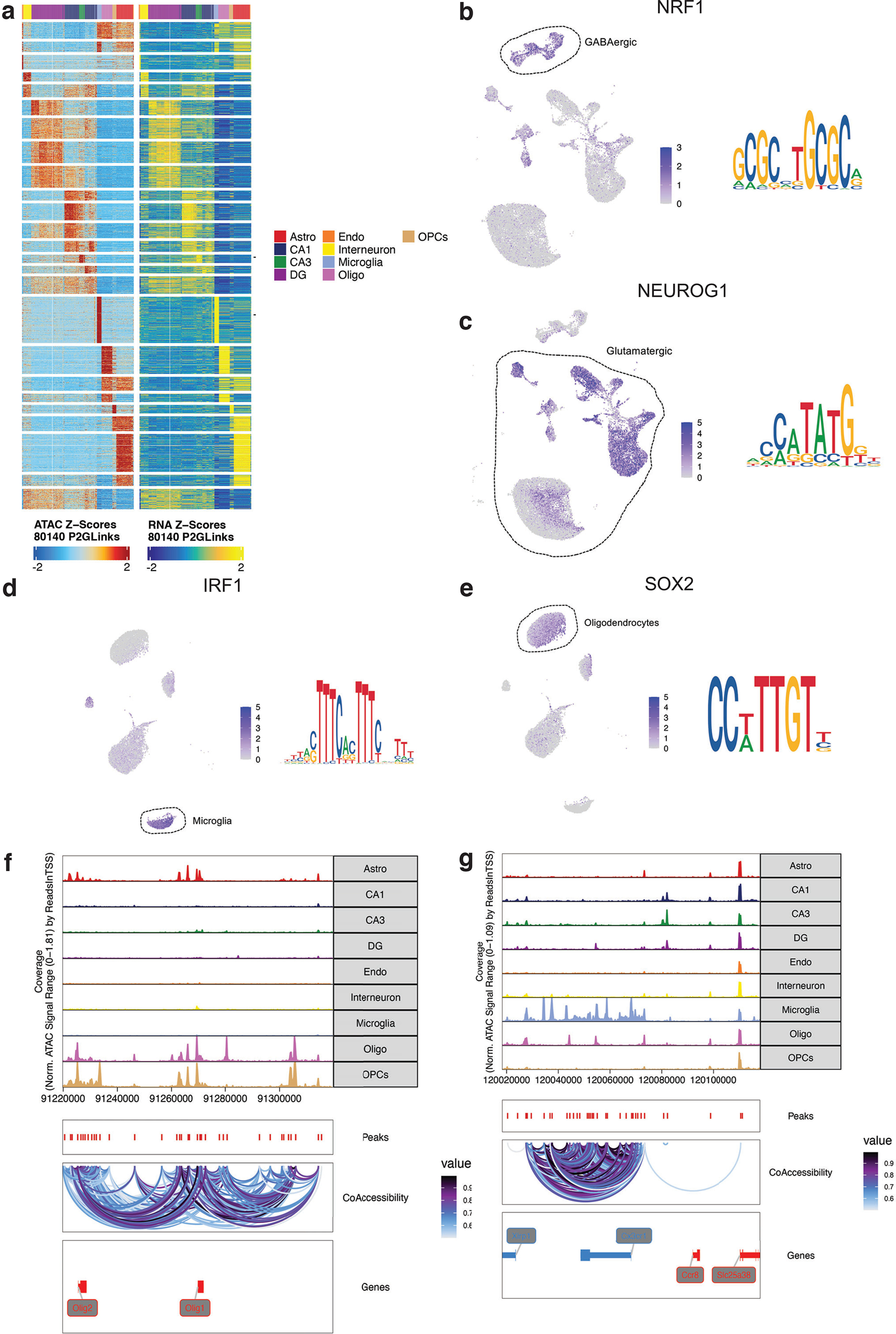
Analysis of TF motifs and differential accessibility from snATAC-seq of the murine hippocampus. ***a***, Integrated analysis of snRNA-seq and snATAC-seq with ArchR peak-to-gene linkage. The heatmap displays k-means clustered genes or chromatin loci as rows. Colored columns indicate cell types. ***b***, Motif feature plot and position weight matrix (PWM) for the TF Nrg1, enriched in GABAergic cells. ***c***, Motif feature plot and PWM for the TF Neurog1, enriched in glutamatergic cells. ***d***, Motif feature plot and PWM for the TF IRF1, enriched in microglia. ***e***, Motif feature plot and PWM for the TF Sox2, enriched in mature oligodendrocytes. ***f***, ArchR-based chromatin co-accessibility analysis of the Olig1 and Olig2 loci. Top panels display genomic tracks of chromatin accessibility, organized by cell type. Bottom panels display location and linkage of co-accessible peaks near the Olig1 and Olig2 loci. ***g***, ArchR-based chromatin co-accessibility analysis of the Cx3cr1 locus. Top panels display genomic tracks of chromatin accessibility, organized by cell type. Bottom panels display location and linkage of co-accessible peaks near the Cx3cr1 locus.

Chromatin organization governs transcription factor (TF) binding and transcriptional regulation, which are necessary for the dynamic adaptation of neural circuits. To complement the cell type-specific transcriptional atlas of the maternal hippocampus, we performed snATAC-seq at identical timepoints to those described above. Our snATACseq dataset includes 38,123 nuclei that pass quality filtering (Fig. 6*a–d*), with an average of 216,990 unique nuclear genome fragments. The proportion of accessible regions within genes, promoters, or distal regulatory sites was 69.6%, 0.1%, 30.3%, respectively. We assigned cell type identity to snATAC-seq clusters using anchoring and label transfer from our snRNA-seq data via Signac (Stuart et al., 2021). Label transfer and integration of snRNA-seq and snATAC-seq demonstrated strong agreement between computational and supervised (marker gene-driven) linkage of the datasets (Fig. 6*e*,*f*). To further correlate transcriptional and chromatin dynamics within cell types, we analyzed the combined datasets using ArchR Peak2GeneLinkage (Granja et al., 2021). We identified strong cell type-specific modules of gene-chromatin correlations, as evidenced by the heatmap in Figure 7*a*, suggesting distinct gene regulatory interactions.

First, given limited existing data on the chromatin landscape of the murine hippocampus, we identified chromatin accessibility signatures for distinct cell types in the mouse hippocampus across all timepoints in a pooled analysis (virgin, E18, postpartum). In the GABAergic neuronal population, we noted the marked increase in the accessibility of the Nuclear Respiratory factor 1 (*NRF-1*) motif relative to glutamatergic neurons (Fig. 7*b*). This TF serves as an activity-dependent activator for the transcription of the β1 subunit of the GABA-A receptor (Z Li et al., 2018). Conversely, we identified the enhanced accessibility of the Neurogenin 1 (*Neurog1*) motif in the glutamatergic population relative to GABAergic neurons (Fig. 7*c*). Neurog1 is a critical TF for glutamatergic fate specification (Reyes et al., 2008; Shaker et al., 2012). In astrocytes relative to oligodendrocytes, there was increased accessibility of the Interferon regulatory factor 1 (*IRF1*) motif (Fig. 7*d*); IRF1 restricts the differentiation of OPCs to oligodendrocytes (Tanner et al., 2011). We also found the accessibility of the Sex determining region Y-box 2 (*SOX2*) motif to be greater in oligodendrocytes (Fig. 7*e*). In murine brain development, the proliferation and differentiation of oligodendrocytes are upregulated by SOX2 expression, and in adulthood, SOX2 facilitates remyelination following white matter damage (S Zhang et al., 2018). These results, while largely expected based on known mechanisms of TF activity in brain cell types, support the fidelity of chromatin accessibility signatures in our dataset.

We then constructed chromatin co-accessibility networks for canonical cell type identity genes for the murine hippocampus, again across all timepoints in a pooled analysis (virgin, E18, postpartum). Co-accessibility is a correlation in chromatin accessibility between two peaks across many single cells. We used ArchR to map proximal and distal chromatin regulatory elements associated with target genes. We identified chromatin co-accessibility in regulatory elements linking Olig1 and Olig2 loci, which are genes predominantly expression in oligodendrocyte lineage cells (Fig. 7*f*). We also mapped the complex *cis*-regulatory landscape surrounding the Cx3cr1 locus, which is associated with microglial identity (Fig. 7*g*).

**Figure 8.**
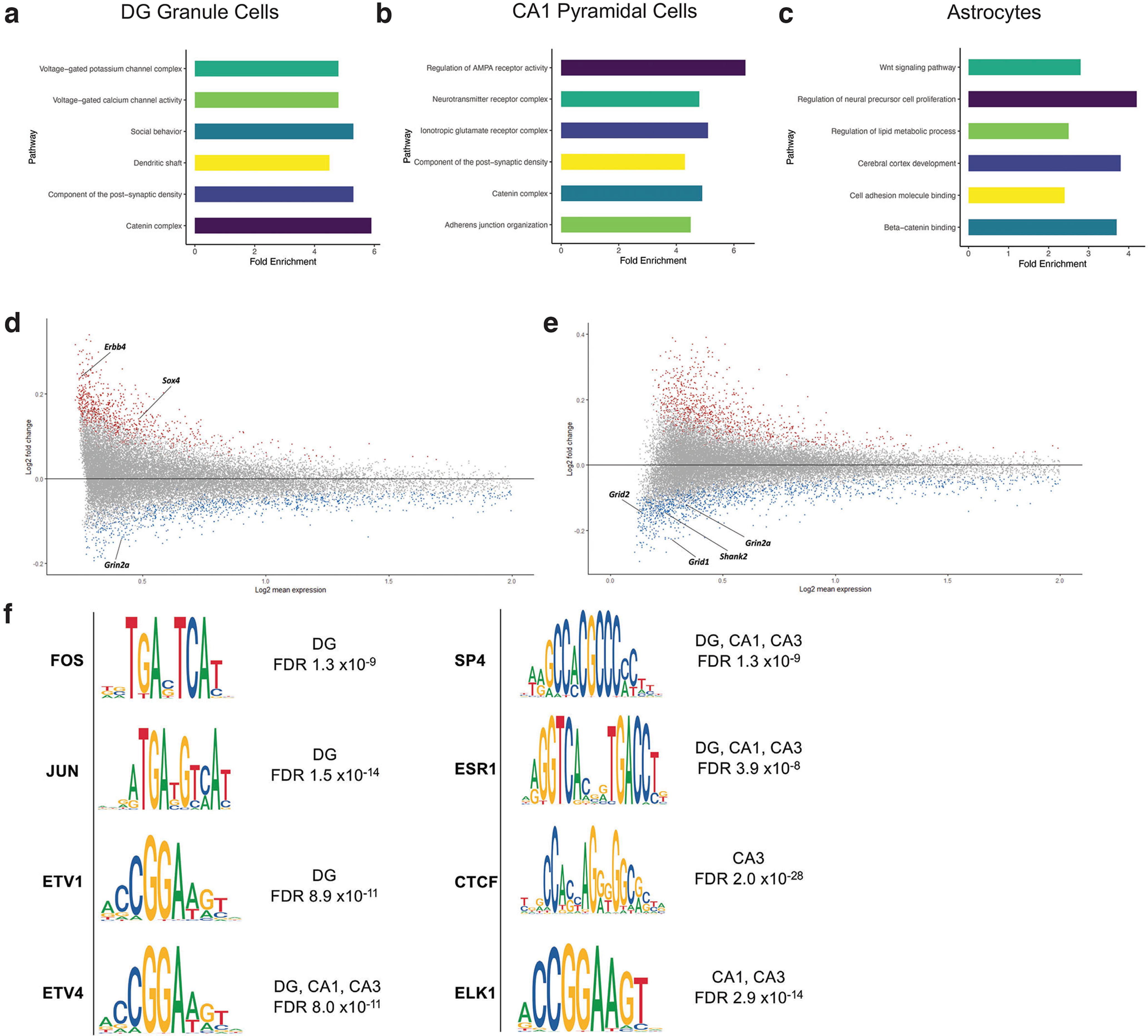
Differential accessibility analysis of snATAC-seq of the murine hippocampus before, during, and after pregnancy. ***a***, GO analysis of differentially accessible peaks (FDR < 5%) between virgin and E18 in DG granule cells. Extended Data Figure 8-1 provides complete results of differential chromatin accessibility analysis, and Extended Data Figure 8-2 provides complete results of GO analysis from snATAC-seq. ***b***, GO analysis of differentially accessible peaks (FDR < 5%) between virgin and E18 in CA1 pyramidal cells. ***c***, GO analysis of differentially accessible peaks (FDR < 5%) between virgin and E18 in astrocytes. ***d***, MA plot (log2 fold change vs log2 mean expression) of differentially accessible peaks in DG comparing virgin versus E18, with select candidates (Erbb4, Sox4, and Grin2a) highlighted. ***e***, MA plot (log2 fold change vs log2 mean expression) of differentially accessible peaks in CA1 comparing virgin versus E18, with select candidates (Grid1, Grid2, Shank2, Grin2a) highlighted. ***f***, Position weight matrices (PWM) for TF motifs with significant changes at E18 compared with virgin. FDR values reflect comparisons in the DG for FOS, JUN, ETV1, ETV4, SP4, and ESR1. FDR values reflect comparisons in CA3 for CTCF and CA1 for ELK1.

We next asked whether there were cell type-specific changes in chromatin accessibility consistent with pregnancy-associated neuroplasticity. We performed pairwise differential accessibility analysis between conditions (virgin, E18, postpartum) within cell types, and these data are provided in Extended Data Figure 8-1. Broadly, using the peaks with significant changes in accessibility during pregnancy (virgin vs E18), we used GO analysis to identify functional pathways that exhibit dynamic cell type-specific changes (DG granule cells, CA1 pyramidal neurons, and astrocytes; Fig. 8*a–c*; Extended Data Fig. 8-2). The Wnt/β-catenin signaling, which is critical in regulating neurogenesis (Arredondo et al., 2020), synapse strength (Okuda et al., 2007), and dendritic structure (Heppt et al., 2020), was a common pathway identified across several cell types and is known to be enhanced by estrogen (Varea et al., 2009). Next, we focused on three themes, similar to the snRNA-seq analysis: neurogenesis, neurotransmission, and structural remodeling.

10.1523/ENEURO.0117-22.2022.f8-1 Extended Data Figure 8-1 Table of differential chromatin accessibility results from snATAC-seq between virgin, E18, and postpartum conditions. Each tab of the file contains results of differential chromatin accessibility analysis in a specific cell type, organized by pairwise comparison between conditions (virgin, E18, and postpartum). The results list the gene of interest, ENSEMBL gene ID, gene biotype, *p*-value, adjusted *p*-value, average log2 fold change, FDR, and DNA coordinates of the loci. Download Figure 8-1, XLS file.

10.1523/ENEURO.0117-22.2022.f8-2 Extended Data Figure 8-2 GO analysis of statistically significant differentially accessible chromatin regions in the virgin versus E18 comparison. Each tab of the file contains results of GO analysis by select cell type. Only statistically significant GO pathways are listed. Download Figure 8-2, XLS file.

#### Neurogenesis

Cell type-specific chromatin accessibility analysis corroborated our transcriptional findings associated with impaired neurogenesis during pregnancy. Specifically, the accessibility of the *Sox4* locus in DG granule cells is decreased during pregnancy (E18) compared with virgin (Fig. 8*d*). Sox4 is a member of the SoxC group within the SOX TF family and facilitates neurogenesis both *in vitro* and *in vivo* (Mu et al., 2012). We further found the accessibility of Erb-B2 receptor tyrosine kinase 4 (*Erbb4*) locus to be lower during pregnancy (E18) compared with virgin (Fig. 8*d*). In the subventricular zone, another neurogenic niche, Erbb4 is necessary for the survival, migration and organisation of NPCs (Anton et al., 2004; H Zhang et al., 2018).

We inferred TF activity through analysis of differential motif accessibility and found evidence to support a decline in neurogenesis during pregnancy. In DG granule cells, we found the decreased accessibility of several FOS and JUN motifs in pregnant mice (E18) relative to virgin controls (Fig. 8*f*). These proteins are members of the Activator protein-1 (AP1) TF family, broadly regulating cell proliferation and differentiation (Shaulian and Karin, 2002). Specific to neurogenesis, the AP1 complex likely regulates NPC proliferation, and Fos is sufficient to rescue NPC self-renewal in SOX2 knock-out (KO) mice (Pagin et al., 2021). Moreover, there is increased accessibility of the Etv1 motif in DG granule cells of pregnant mice (E18) when compared with virgin controls (Fig. 8*f*). *In vitro*, ETV1 represses cell proliferation genes in murine cerebellar granule cells and promotes mature neural circuit formation (Okazawa et al., 2016).

#### Structural remodeling

We identified changes in cell type-specific chromatin accessibility to support the structural remodeling of the neural circuit associated with pregnancy. In particular, in CA3 pyramidal neurons, we discovered increased accessibility of the Nectin Cell Adhesion Molecule 3 (*Nectin3*) locus during pregnancy (E18) relative to virgin conditions. This synaptic cell adhesion molecule is located at the postsynaptic terminal and coordinates the actin cytoskeleton and cadherin-catenin complex during synaptogenesis and synaptic remodeling (R Liu et al., 2019). Likewise, Shank2 is a postsynaptic terminal component and binding partner of PSD-95 (Brandstätter et al., 2004). By snRNA-seq, we found the increased expression of PSD-95 in CA1 pyramidal neurons during pregnancy (E18) compared with virgin. Interestingly, we also noted that the accessibility of the *Shank2* locus is greater in CA1 pyramidal neurons during pregnancy (E18) compared with virgin (Fig. 7*b*). Akin to PSD-95, Shank2 is a postsynaptic scaffold protein localized to excitatory synapses and, along with several other components, organizes the location of metabotropic and ionotropic glutamate receptors (Monteiro and Feng, 2017).

With regard to TF motifs, we identified increased accessibility of the ETV4 motif in DG granule cells, as well as CA1 and CA3 pyramidal neurons, during pregnancy (E18) relative to virgin conditions (Fig. 8*f*). This TF likely mediates BDNF signaling and is highly expressed during hippocampal dendrite development, with ETV4 deletion resulting in decreased dendritic complexity and size (D Liu et al., 2016; Fontanet et al., 2018). We further identified increased accessibility of the SP4 TF motif in CA1 and CA3 pyramidal neurons during pregnancy (E18) compared with virgin (Fig. 7*c*). In rodents, SP4 has been found to modulate dendritic arborization and pruning as well as NMDAR trafficking (Ramos et al., 2007; Sun et al., 2015). Notably, the accessibility of the Estrogen receptor α (ESR1) motif is increased during pregnancy (E18) in DG granule cells and CA1 and CA3 pyramidal neurons compared with virgin (Fig. 8*f*). In the hippocampus, this nuclear receptor modulates a broad spectrum of plasticity, including dendritic spine density and the abundance of NMDARs (Mukai et al., 2010). This finding is consistent with a hormone-mediated model of pregnancy-associated neuroplasticity.

#### Neurotransmission

Changes in the chromatin accessibility landscape during pregnancy also corroborate pregnancy-associated adaptations in neurotransmission. In CA1 pyramidal neurons, there is increased accessibility of the loci for glutamate receptor δ-1 and δ-2 subunits (*Grid1* and *Grid2*) during pregnancy (E18) compared with virgin (Fig. 8*e*). Both genes play a role in synaptic plasticity, albeit in different neuronal populations (Fossati et al., 2019) and Grid1, in particular, mediates hippocampal mGlu5 signaling (Suryavanshi et al., 2016). Furthermore, in CA1 pyramidal neurons and DG granule cells, we identified increased accessibility of the NMDAR subunit ε−1 (*Grin2a*) locus (Fig. 7*b*) in E18 compared with control. In CA3 pyramidal neurons, we identified the NMDAR subunit ε−2 (*Grin2b*) locus as more accessible at E18 compared with virgin. These subunits are integral to synaptic plasticity, with Grin2a mutant mice experiencing reduced LTP in CA1 (Sakimura et al., 1995), and in Grin2b mutants, NMDAR-mediated LTD is eliminated (Brigman et al., 2010).

Analysis of differential TF motifs between pregnancy conditions revealed further evidence of pregnancy-associated modifications in neurotransmission. The motif for CCCTC-binding factor (CTCF) is more accessible in CA3 pyramidal neurons of the pregnant hippocampus (E18) when compared with virgin mice (Fig. 8*f*). CTCF-induced gene expression is thought to underlie adult hippocampal memory via chromatin reorganization, and CTCF KO mice show disrupted LTP (Sams et al., 2016). We also noted the enhanced accessibility of the ETS TF ELK1 in CA3 and CA1 pyramidal neurons during pregnancy (E18) when compared with virgin (Fig. 8*f*). In the hippocampus, ELK1 phosphorylation via the MAPK/ERK pathway has been associated with induction of LTD and is also thought to mediate LTP-dependent gene regulation (Davis et al., 2000; Besnard et al., 2011).

In summary, our multiomics characterization provides a detailed landscape of complementary layers of regulatory control governing peripartum plasticity and identify candidate regulators of hormone-mediated adaptation.

## Discussion

Despite long-standing recognition of the extraordinary plasticity of the female brain during and after pregnancy, there is a shortage of molecular insights into this physiological adaptation. Hormone-driven plasticity during pregnancy has likely evolved to prepare females for child-rearing and improve offspring survival (Brunton and Russell, 2008). Human neuroimaging studies have demonstrated pregnancy-associated decreases in gray matter volume that persists for at least two years postpartum (Hoekzema et al., 2017). These volume reductions are particularly prominent in regions implicated in maternal caregiving (Barha and Galea, 2017; Michaelian et al., 2019). Although the existing literature on humans is limited, pregnancy-associated cognitive changes are supported by self-reported symptoms of working memory deficits and general “mental fogginess” (Brown and Schaffir, 2019). Globally, >200 million women are pregnant each year, and it is therefore critical that we understand the mechanisms by which pregnancy shapes the female brain.

In this study, we leverage snRNA-seq and snATAC-seq to identify candidate transcriptional and epigenetic regulators of pregnancy-associated neuroplasticity. We focus on the hippocampus, as this region demonstrates substantial plasticity across the mammalian lifespan, and the DG is one of only two known areas in which neurogenesis occurs in adult mammals (Cameron and Mckay, 2001; Gould, 2007). We identify numerous potential candidate regulators of neurogenesis, neurotransmission and structural remodeling during and after pregnancy, with the most dramatic changes occurring between virgin (prepregnancy) and E18. These results are broadly consistent with previous *in vitro* and *in vivo* experiments demonstrating a marked impact of sex hormones on neural plasticity. Specifically, in mice, late pregnancy is associated with reduced neurogenesis in the DG that continues into the early postpartum period (Rolls et al., 2008; Kim et al., 2010). Pregnancy also correlates with increased dendritic spine density in the CA1 neurons of female rats (Kinsley et al., 2006; Pawluski and Galea, 2006), as well as changes in GABAergic neurotransmission in mice during pregnancy (Maguire et al., 2009).

Periods of intense neuroplasticity are often associated with vulnerability for mental illness, as perturbations during so-called “critical periods” can have profound long-term consequences for cognition, memory, and behavior. Therefore, it is not surprising that the peripartum period is also marked by increased incidence of psychiatric illness, including depression, anxiety, and psychosis (Galea et al., 2014; Barba-Müller et al., 2019). Future work should characterize how stress or other perturbations during pregnancy modulate peripartum neuroplasticity, as well as cell type-specific regulators of maternal behavior. For example, in pregnant rats, stress during gestation results in increased neurogenesis and also impacts hippocampal glucocorticoid receptor density (Pawluski et al., 2015).

There are likely other drivers of neuroplasticity during pregnancy that require deeper investigation beyond this study. In particular, immune adaptation across gestation is critical to safeguard the mother from infection and prevent an immune reaction directed at the fetus (Peterson et al., 2020). While classically implicated in maternal-fetal tolerance, T cells also play a role in adult murine neurogenesis, and pregnant mice lacking a T cell population only display a marginal decline in neurogenesis during pregnancy (Ziv et al., 2006; Rolls et al., 2008). Interestingly, the pregnancy-associated decrease in neurogenesis is reversed following T cell restoration in nude mice (Rolls et al., 2008).

The findings of the present study should be interpreted in the context of several technical and analytical limitations. Importantly, changes in the maternal brain are highly species-dependent, and therefore, the results of this study must be interpreted in the context of the murine brain. snRNA-seq suffers from low capture efficiency and gene dropout, which may bias the analysis toward more highly expressed genes. Similarly, chromatin accessibility from snATAC-seq is sparse, and it is challenging to infer precise biological function from changes in accessible regions and putative gene regulatory networks. The comparisons between conditions are pairwise, which limits any conclusion about the precise trajectory across all three peripartum timepoints. While we identified changes between groups that were statistically significant (FDR < 5%), the fold changes are often modest, at best, which may reflect a limitation of the single-cell sequencing approach. Despite these limitations, this dataset is an important resource for mapping cell type-specific adaptation to the unique context of pregnancy.

## Synthesis

Reviewing Editor: Michael Michaelides, NIDA-NIH Decisions are customarily a result of the Reviewing Editor and the peer reviewers coming together and discussing their recommendations until a consensus is reached. When revisions are invited, a fact-based synthesis statement explaining their decision and outlining what is needed to prepare a revision will be listed below. The following reviewer(s) agreed to reveal their identity: Randall Ellis.

Reviewer 1

The single-nuclei RNA-seq and ATAC-seq datasets will be valuable to many scientific fields (hippocampus, memory, development, pregnancy, etc.), and the authors should be commended for providing a great resource. My issues with the manuscript are minor and have to do with 1) Lack of detail about the cell type classification procedures used, and 2) Shallowness of results reporting.

1) In the section, “Identification of cell types in snRNA-seq,” the manuscript describes a procedure wherein classifiers are used to predict cell type identities in the hippocampus using data from the Allen Brain Atlas. While the performance reported is high (area under the receiver operating characteristic curve > 0.95), a lot details are omitted for these experiments.

First, “ROC” is not defined, and this should presumably be “AUROC” (adding “area under” in front of “receiver operating characteristic”).

Next, there is no description of how the data were split between training, validation, and test sets. Was it an 80/20 split for train/test, with no validation set? Was there a cross-validation procedure used to choose specific model hyperparameters? If a cross-validation procedure was used, there should also be a completely held out test set that is not used as part of the cross-validation experiments to ensure the models are generalizing beyond the training and validation sets.

Third, this section mentions data pre-processing, which can carry a risk of data leakage. That is, if the pre-processing steps are in any way impacted by the validation or test sets (e.g., averaging over all cells instead of just the training set cells as part of an expression normalization procedure), this would represent data leakage, a big concern in machine learning research, and could inflate the performance. As a more complete example, let’s say you split the data into just training and test sets. If you were z-scoring the data, you would only want to z-score the training set, then use the means and standard deviations from the training set to z-score the test set. This would ensure that no information is being leaked from the test set during data pre-processing. Data leakage is a big problem in machine learning research and it would be great if this highly valuable and relevant study ensures it has not occurred.

Fourth, the text doesn’t specify which classification scenarios showed AUROC>0.95. The text states that a “one vs all” approach was used, so these experiments are predicting Pvalb vs. all other cells, as one example. Did the chosen models show ROC>0.95 for all cell types in all tissues? Were some tissues and cell types better predicted than others? Was the prediction performance a function of the number of cells in a certain population? All of this information would be valuable to have in the manuscript or Supplement.

Lastly, there’s an instance of “wass” that should presumably be “was.”

2) The results in the paper are very valuable, but are a bit too descriptive and surface-level, without much synthesis. The primary results are the reporting of individual differentially expressed/accessible genes/regions that have to do with relevant biological processes, with accompanying references. While these results are valuable, I think the manuscript could delve deeper and use other approaches to extract important results. Instead of mainly relying on individual genes, more data could be shown on pathways, ontologies, networks, and larger genomic structures.

One recommendation would be to use a network analysis tool such as WGCNA or MEGENA, and see how the genes (in RNA-seq; could also do the ATAC-seq) co-express, what modules can be drawn from their co-expression and their functional annotations, the hub genes in these modules, and whether the hubs are differentially expressed. This would elevate the article from talking about individual genes and their functions, to networks of genes and their coordinated expression within specific tissues/cell types, along with function.

Related to this, please show ontology analyses for the RNA-seq data, which is already presented for the ATAC-seq data. These analyses also help readers think about pathways and larger biological structures, rather than just individual genes.

Another recommendation is to try to synthesize the RNA-seq and ATAC-seq datasets. Do they agree with one another? As in, are there differentially accessible loci that correspond to differentially expressed genes, and does the accessibility agree with the expression (e.g., increased accessibility with increased expression)? Even if different nuclei were used for RNA-seq and ATAC-seq, these analyses can still be done. There are many multi-omic methods that aim to synthesize datasets for subtyping populations and biomarker prediction (e.g., the three pregnancy timepoints), some of which are discussed at the following reference:

Multi-omics Data Integration, Interpretation, and Its Application (2020). Indhupriya Subramanian, Srikant Verma, Shiva Kumar, Abhay Jere and Krishanpal Anamika

Overall, the study is excellent and a major contribution, but the two remaining challenges are 1) Elaborating on the details of the machine learning experiments, and 2) Synthesizing the genomics results without relying on reporting changes in individual genes, and reporting the results in terms of larger structures (pathways, ontologies, networks).

Reviewer 2

For this murine study the authors performed single nuclei RNA-seq and compared gene expression on hippocampi from 8 week virgin mice, pregnant at E18 mice and 3 weeks post-partum mice. They also performed single nuclei ATAC-seq and compared accessible genes between the same groups of mice. They identified differentially expressed genes that could be grouped into those affecting neurogenesis, those affecting neurotransmission and those affecting dendritic spines and synapses. They also identified accessible genes that could be grouped similarly. Although this study has the potential to provide mechanistic insight into pregnancy associated neuroplasticity through differential transcription of genes and epigenetically regulated transcription of genes, there was no attempt to validate any differentially expressed genes. Finally, specific results were not really integrated into the Discussion.

Methods details - number of reads?

Results - Please include data only in Results and reserve contextualization of data within the existing literature for the Discussion.

An important question for reporting these results is, what is the significance of differences compared to virgin as opposed to 3 weeks post-partum? For example, if a difference is noted between E18 and virgin but not between E18 and post-partum, does this mean that differences are sustained? If differences are found between E18 and post-partum, does this mean that there were not differences between virgin and E18?

Figure 1E - UMAP for E18, PP and Virgin looking extremely similar

Figure 2 - legend incorrectly indicates C as violin plots - should be B; A) how does representation of CA1, DG and CA3 inform us?; C) representation of glutamatergic cells inform us D) how does representation of GABAergic cells and are these cells from CA1, DG and CA3 all pooled together?; would looking at hippocampal regions separately be informative?

Figure 3 - Separate representation of hippocampal regions from cell types on strip plots. Group volcano plots for hippocampal regions and for cell types.

Figure 4 - Separate out CA1 and CA3 in chord diagrams. Label the figure as upregulated and downregulated.

Figure 5 - Why is DG included as a cell type?

Discussion - Needs more synthesis of specific RNAseq and ATACseq findings. Address the importance of differential expression in DG versus CA1 versus CA2.

## Author Response

Dear Reviewers,

Thank you for your thoughtful review of our manuscript “The Genomic Architecture of Pregnancy-Associated Plasticity in the Maternal Mouse Hippocampus.” The reviewers’ comments were very helpful in improving the quality of the manuscript. We have made substantial revisions to the analyses and manuscript in response to the review. We have outlined our responses to the reviewer comments below.

Reviewers’ Comments and Point by Point Response:

Reviewer 1

The single-nuclei RNA-seq and ATAC-seq datasets will be valuable to many scientific fields (hippocampus, memory, development, pregnancy, etc.), and the authors should be commended for providing a great resource. My issues with the manuscript are minor and have to do with 1)

Lack of detail about the cell type classification procedures used, and 2) Shallowness of results reporting.

1) In the section, “Identification of cell types in snRNA-seq,” the manuscript describes a procedure wherein classifiers are used to predict cell type identities in the hippocampus using data from the Allen Brain Atlas. While the performance reported is high (area under the receiver operating characteristic curve > 0.95), a lot details are omitted for these experiments.

First, “ROC” is not defined, and this should presumably be “AUROC” (adding “area under” in front of “receiver operating characteristic”).

Response: We have revised the text to state AUROC instead of ROC, with appropriate definition of the abbreviation.

Next, there is no description of how the data were split between training, validation, and test sets. Was it an 80/20 split for train/test, with no validation set? Was there a cross-validation procedure used to choose specific model hyperparameters? If a cross-validation procedure was used, there should also be a completely held out test set that is not used as part of the cross-validation experiments to ensure the models are generalizing beyond the training and validation sets.

Response: We have revised the Methods and Results to provide clarity on the data training and test sets. For each cell type annotated in the Allen Brain Atlas, we trained a separate one vs all Support Vector Machine (SVM) model with a Radial Baisis Function kernel. Prior to training, the expression matrices were centered, scaled, and normalized in the same fashion as our dataset.

Models were trained using a 5-fold cross validation approach and only models with AUROC > 0.95 were used. Using each of the models, we then predicted the cell types of each cell in our dataset. Only cells with in-class prediction (e.g. CA1 vs all others) probability >0.95 were retained.

Third, this section mentions data pre-processing, which can carry a risk of data leakage. That is, if the pre-processing steps are in any way impacted by the validation or test sets (e.g., averaging over all cells instead of just the training set cells as part of an expression normalization procedure), this would represent data leakage, a big concern in machine learning research, and could inflate the performance. As a more complete example, let’s say you split the data into just training and test sets. If you were z-scoring the data, you would only want to z-score the training set, then use the means and standard deviations from the training set to z-score the test set. This would ensure that no information is being leaked from the test set during data pre-processing. Data leakage is a big problem in machine learning research and it would be great if this highly valuable and relevant study ensures it has not occurred.

Response: We thank the Reviewer for this important point, as we agree regarding the importance of preventing data leakage in machine learning applications. The training data from the Allen Brain Atlas was pre-processed independently, but using identical metrics. Therefore, data leakage should not be a concern. The independent nature of the pre-processing has been clarified in the text.

Fourth, the text doesn’t specify which classification scenarios showed AUROC>0.95. The text states that a “one vs all” approach was used, so these experiments are predicting Pvalb vs. all other cells, as one example. Did the chosen models show ROC>0.95 for all cell types in all tissues? Were some tissues and cell types better predicted than others? Was the prediction performance a function of the number of cells in a certain population? All of this information would be valuable to have in the manuscript or Supplement.

Response: The cell type prediction for all cell types demonstrated AUROC>0.95. The model demonstrated slightly lower sensitivity and specificity (but still >95%) for less abundant cell populations (e.g. Sncg positive cells). This data has been added as Table 1-1.

Lastly, there’s an instance of “wass” that should presumably be “was.”

Response: We have corrected the typographical error identified by the Reviewer.

2) The results in the paper are very valuable, but are a bit too descriptive and surface-level, without much synthesis. The primary results are the reporting of individual differentially expressed/accessible genes/regions that have to do with relevant biological processes, with accompanying references. While these results are valuable, I think the manuscript could delve deeper and use other approaches to extract important results. Instead of mainly relying on individual genes, more data could be shown on pathways, ontologies, networks, and larger genomic structures.

One recommendation would be to use a network analysis tool such as WGCNA or MEGENA, and see how the genes (in RNA-seq; could also do the ATAC-seq) co-express, what modules can be drawn from their co-expression and their functional annotations, the hub genes in these modules, and whether the hubs are differentially expressed. This would elevate the article from talking about individual genes and their functions, to networks of genes and their coordinated expression within specific tissues/cell types, along with function.

Related to this, please show ontology analyses for the RNA-seq data, which is already presented for the ATAC-seq data. These analyses also help readers think about pathways and larger biological structures, rather than just individual genes.

Another recommendation is to try to synthesize the RNA-seq and ATAC-seq datasets. Do they agree with one another? As in, are there differentially accessible loci that correspond to differentially expressed genes, and does the accessibility agree with the expression (e.g., increased accessibility with increased expression)? Even if different nuclei were used for RNA-seq and ATAC-seq, these analyses can still be done. There are many multi-omic methods that aim to synthesize datasets for subtyping populations and biomarker prediction (e.g., the three pregnancy timepoints), some of which are discussed at the following reference:

Multi-omics Data Integration, Interpretation, and Its Application (2020). Indhupriya Subramanian,

Srikant Verma, Shiva Kumar, Abhay Jere and Krishanpal Anamika

Overall, the study is excellent and a major contribution, but the two remaining challenges are 1)

Elaborating on the details of the machine learning experiments, and 2) Synthesizing the genomics results without relying on reporting changes in individual genes, and reporting the results in terms of larger structures (pathways, ontologies, networks).

Response:

In terms of the agreement between snRNA-seq and snATAC-seq, we have now included metrics for the label transfer and integration of the two datasets in the Results section. We found excellent concordance between computationally-predicted and manually-annotated cell type identification of snATAC-seq. This data is now shown in Figure 6F-G. We have included gene ontology analysis for the snRNA-seq in the Results section and Figure 3.

To further develop the snATAC-seq dataset, we used the analysis package ArchR to analyze chromatin co-accessibility networks. Co-accessibility is a correlation in accessibility between two peaks across many single cells. We identified cell type-specific co-accessibility networks in the hippocampus. This analysis has been added to the Results and Figure 7.

To synthesize the RNA-seq and ATAC-seq datasets, we used the ArchR Peak2GeneLinkage approach. While chromatin co-accessibility, described above, looks for correlations in peaks in snATAC-seq data, peak-to-gene linkage leverages integrated snRNA-seq and snATAC-seq datasets to identify correlations between peak accessibility and gene expression. These links may suggest gene regulatory interactions. We have included a heatmap of hierarchically clustered gene-chromatin correlations in Figure 7. Overall, we identified strong cell type-specific correlation between RNA and ATAC signal.

Reviewer 2

Methods details - number of reads?

Response: The number of reads per nucleus for snRNA-seq and snATAC-seq have been added to the Methods section.

Results - Please include data only in Results and reserve contextualization of data within the existing literature for the Discussion.

Response: We have revised the Results section to include more data synthesis, and moved some contextualization to the Discussion. However, we have retained some of the context for specific genes, as we feel this is necessary in order to understand the gene expression and chromatin accessibility results.

An important question for reporting these results is, what is the significance of differences compared to virgin as opposed to 3 weeks post-partum? For example, if a difference is noted between E18 and virgin but not between E18 and post-partum, does this mean that differences are sustained? If differences are found between E18 and post-partum, does this mean that there were not differences between virgin and E18?

Response: The primary reasoning of sampling three time points is that there are dynamic changes in the brain between ‘non-pregnant’ (virgin), pregnant, and post-partum states. These reflect not only the changes in sex hormones, but also the post-natal adaptation to rearing offspring, as exposure to offspring is known to trigger adaptation in the maternal brain (e.g. via oxytocin, as well as other less poorly defined mechanisms). Importantly, all comparisons are pairwise, and this is a critical limitation of the available methods. As such, it is difficult to disentangle whether differences that are significant in one comparison but not another are biological or technical. We have now acknowledged this limitation in the Discussion. We have been careful to note in the Results which comparison is statistically significant, and the results of all comparisons by cell type are in the Supplemental Tables. It is not surprising that some changes are sustained from virgin to postpartum, while others are transient during pregnancy.

We would hypothesize that changes that are only found in the virgin vs postpartum comparison might represent more chronic changes in response to offspring exposure, as opposed to peak estrogen exposure during pregnancy, but validation of specific candidates of interest would be necessary to confidently make that conclusion. Therefore, we have softened our interpretation, where appropriate, given this limitation.

Figure 1E - UMAP for E18, PP and Virgin looking extremely similar:

Response: The Reviewer is correct. The purpose of showing this figure is to demonstrate that the cell clustering via UMAP is nearly identical between the three conditions. In some experiments, the UMAP can appear different as new cell types/states arise, or if there are significant batch effects between samples. This is not the case in our dataset. Global cell states remain the same between the three conditions, but gene expression within cell types changes during the peripartum period. This has been clarified in the text.

Figure 2 - legend incorrectly indicates C as violin plots - should be B; A) how does representation of CA1, DG and CA3 inform us?; C) representation of glutamatergic cells inform us D) how does representation of GABAergic cells and are these cells from CA1, DG and CA3 all pooled together?; would looking at hippocampal regions separately be informative?:

Response: The typographical error in the legend for Figure 2 has been corrected. The reviewer brings up an important point of distinguishing cell type from cell location/region in the hippocampus, which may have led to confusion. To clarify this in the figures and text, we have changed the terminology and labeling of the cell types. We now employ the terms “Dentate

Gyrus (DG) Granule Cells", “CA1 Pyramidal Cells” and “CA3 Pyramidal Cells”. This is consistent with previously published single cell resources of the mouse hippocampus (https://hipposeq.janelia.org/). In our dataset, we were unable to distinguish nuclei from dorsal/ventral sub-regions of the hippocampus.

Figure 3 - Separate representation of hippocampal regions from cell types on strip plots. Group volcano plots for hippocampal regions and for cell types.

Response: As above, we have clarified the labeling of cell types to avoid confusion between region and cell type. With this important clarification, we believe the present organization of the figures is logical.

Figure 4 - Separate out CA1 and CA3 in chord diagrams. Label the figure as upregulated and downregulated.

Response: All of the cell types are displayed in the chord diagrams simultaneously, as the purpose is to display cell communication (ligand-receptor interactions). We have, however, labeled the figure as “upregulated” and “downregulated” as the Reviewer suggests.

Figure 5 - Why is DG included as a cell type?

Response: As above, we have clarified that we are analyzing granule cells from dentate gyrus.

Discussion - Needs more synthesis of specific RNAseq and ATACseq findings. Address the importance of differential expression in DG versus CA1 versus CA2.

Response: Consistent with the recommendations of Reviewer 1, we have added several elements to the manuscript that synthesize and expand upon combined analysis of RNA and ATAC-seq findings. Specifically, we have now included the following:

To further develop the snATAC-seq dataset, we used the analysis package ArchR to analyze chromatin co-accessibility networks. Co-accessibility is a correlation in accessibility between two peaks across many single cells. We identified cell type-specific co-accessibility networks in the hippocampus. This analysis has been added to the Results and Figure 7. A complete table of co-accessibility results are included as Table 7-1.

To synthesize the RNA-seq and ATAC-seq datasets, we used the ArchR Peak2GeneLinkage approach. While chromatin co-accessibility, described above, looks for correlations in peaks in snATAC-seq data, peak-to-gene linkage leverages integrated snRNA-seq and snATAC-seq datasets to identify correlations between peak accessibility and gene expression. These links may suggest gene regulatory interactions. We have included a heatmap of hierarchically clustered gene-chromatin correlations in Figure 7. Overall, we identified strong cell type-specific correlation between RNA and ATAC signal.
